# On Chronic Vomiting, as Symptomatic of Disease of the Kidneys

**Published:** 1851-11

**Authors:** 


### PATHOLOGY AND PRACTICE OF MEDICINE.

*On Chronic Vomiting, as symptomatic of Disease of the Kidneys.*—Dr. Cathcart Lees, of the Meath Hospital, Dublin, reports in the Dublin Quarterly Journal of Medical Science, for August, 1851, several cases illustrative of the connection between chronic vomiting and disease or irritation of the kidneys. He remarks:—

"Nausea and vomiting are very constant occurrences towards the latter periods of chronic degeneration of the kidneys, but as they are met with in most cases of irritation of the organ, no matter from what cause, whether it be simple acute nephritis, the mechanical irritation of a calculus, or gradual obstruction of the tubes from the fat, or any other form of deposit, we cannot establish any positive diagnosis from its occurrence. As to any degree of certainty with regard to even the seat of the disease, derivable from peculiarities in the times of vomiting, or in the nature of the matters vomited, I am not sufficiently prepared to speak positively. In two of these cases the patients always vomited early in the morning, previously to getting up, or to the taking of any food. I at one time thought that this might be a means of diagnosing vomiting depending on disease of the kidney from other forms of vomiting, but further experience has satisfied me that this rule does not hold good. We must, therefore, take into consideration all the other circumstances of the case, the history, age, sex of the patient, and particularly the situations to which pain is referred; but even with the most careful examination we shall often be puzzled, particularly in cases where we suspect the brain may be the cause of the vomiting, and yet where the character of the pulse (as occasionally happens) does not afford us sufficient indications, nor have we any other symptoms referable to it, or indeed to any organ in particular, to account for the frequent vomiting."

"As to the treatment of these cases of chronic vomiting, if it occur early in the morning, when the patient is fasting, I have found the most successful plan to be that of making him take some light kind of food before getting up, remaining quiet for a short time afterwards. If it occurs at an early period of the disease, and particularly if pain in the loins is complained of, or even a sensation of weight, three or four ounces of blood taken, by cupping, from that region, affords great relief. I have seen, in some cases, most satisfactory results from the use of equal parts of boiled milk and lime water, given frequently during the day, particularly when diarrhœa was present. In other cases, one, two, or three drops of hydrocyanic acid, combined with three grains of bicarbonate of soda, repeated every three hours, and given immediately before food or drink is taken, proves useful. Creasote, in doses of one or two drops, frequently repeated, is also often of service. In one very bad case, lately admitted into the Meath Hospital, that of a young girl laboring under general anasarca, with constant vomiting and severe diarrhœa, much benefit was derived from the liquor ferri pernitratis, in five-drop doses, four times a day. Occasionally a blister over the stomach, a little brandy, or from twenty to thirty drops of Hoffman's anodyne liquor, will check the vomiting. Some practitioners have recommended the use of opium, but I have not used it, in consequence of the tendency to head symptoms which exists in all cases where the structure of the kidneys is deranged; and as, in many instances, it is difficult to decide whether the symptoms are caused by mere mechanical irritation, or obstruction in the substance of the kidney itself, or whether they depend on the general poisoning of the system, from the retention and circulation in the blood of urea and other excrementitious substances, which ought to pass out of the system with the urine."

